# The genetic evolution of canine parvovirus – A new perspective

**DOI:** 10.1371/journal.pone.0175035

**Published:** 2017-03-31

**Authors:** Pei Zhou, Weijie Zeng, Xin Zhang, Shoujun Li

**Affiliations:** 1 College of Veterinary Medicine, South China Agricultural University, Tianhe District, Guangzhou, Guangdong Province, People’s Republic of China; 2 Key Laboratory of Comprehensive Prevention and Control for Severe Clinical Animal Diseases of Guangdong Province, Guangzhou, Guangdong Province, People’s Republic of China; 3 Guangdong Engineering and Technological Research Center for Pets, Guangzhou, Guangdong Province, People’s Republic of China; Sun Yat-Sen University, CHINA

## Abstract

To trace the evolution process of CPV-2, all of the VP2 gene sequences of CPV-2 and FPV (from 1978 to 2015) from GenBank were analyzed in this study. Then, several new ideas regarding CPV-2 evolution were presented. First, the VP2 amino acid 555 and 375 positions of CPV-2 were first ruled out as a universal mutation site in CPV-2a and amino acid 101 position of FPV feature I or T instead of only I in existing rule. Second, the recently confusing nomenclature of CPV-2 variants was substituted with a optional nomenclature that would serve future CPV-2 research. Third, After check the global distribution of variants, CPV-2a is the predominant variant in Asia and CPV-2c is the predominant variant in Europe and Latin America. Fourth, a series of CPV-2-like strains were identified and deduced to evolve from modified live vaccine strains. Finally, three single VP2 mutation (F267Y, Y324I, and T440A) strains were caught concern. Furthermore, these three new VP2 mutation strains may be responsible for vaccine failure, and the strains with VP2 440A may become the novel CPV sub-variant. In conclusion, a summary of all VP2 sequences provides a new perspective regarding CPV-2 evolution and the correlative biological studies needs to be further performed.

## Introduction

Canine parvovirus (CPV) belongs to the genus *Protoparvovirus* and the family *Parvoviridae* and causes a highly contagious and fatal disease in dogs [1]. CPV is a non-enveloped DNA virus with an approximately 5000-nucleotide, single-stranded DNA genome containing two open reading frames (ORFs). The first ORF encodes two non-structural proteins, NS1 and NS2. The second ORF encodes two structural proteins, VP1 and VP2 [2]. VP1 and VP2 each encode parts of the viral capsid, which is assembled from 54 copies of VP2 and 6 copies of VP1 [3]. VP2, the major capsid protein, is also the major antigenic protein and determines viral tissue tropism and host range [4, 5]. NS1, a pleiotropic nuclear phosphoprotein, plays an essential role in viral replication and is responsible for inducing cell apoptosis [6, 7].

Although CPV is a DNA virus, its genomic substitution rate is approximately 10^−4^ per site per year, which is similar to RNA viruses [8]. In 1978, CPV-2 was first identified from outbreaks in canines in the United States and Australia [9, 10]; it was then reported in many countries during 1978 and 1979 [11]. CPV-2 is closely related to feline parvovirus (FPV); therefore, it is assumed to be a host variant of FPV [12, 13]. CPV-2 was named to distinguish and differentiate it from an unrelated canine minute virus (CPV-1) [14]. In the 1980s, the original CPV-2 type was completely replaced by two new antigenic variants in canines, which were termed CPV types 2a (CPV-2a) and 2b (CPV-2b) [15, 16]. In 2000, CPV-2c with Asp426Glu substitution was reported in Italy[17]. With its antigenicity constantly drifting, an increasing number of further mutations of VP2 have been described, and various viral mutants have been named [17–20]. However, the nomenclature of these variants is inconsistent and confusing.

To distinguish the nomenclature and to further understand CPV-2 evolution, the sequence characteristics of the VP2 gene of CPV-2 and FPV were updated in this study.

## Materials and methods

### Sequence data

By 17^th^ June 2016, 2170 VP2 sequences of CPV-2 and 245 sequences of FPVwere downloaded from the NCBI (from 1978 to 2015). Among the 2170 sequences of CPV-2, 1679 have collection date and 491 not have collection date. Among the 245 sequences of FPV, 170 have collection date and 75 not have collection date. 1679 sequences of CPV-2 were ordered according to the collection date from 1978 to 2015 by using Microsoft Excel.

### Sequence analysis

The sequences that have collection date were used for mutation analysis. The nucleotide alignments were performed using BioEdit and the nucleotide substitution were manually edited by checking 50 sequences every time. Then, the amino acid (aa) mutation was summarized according to the nucleotide substitution.

### Graph

The aa mutational figs were created using the Prism 5.0 software (GraphPad Software). The VP2 structure was downloaded from SWISS-MODEL (https://swissmodel.expasy.org/), and the aa residues were depicted in PyMOL (Version 1.5.0.4).

## Results

### The VP2 555 and 375 aa of CPV-2 may need rule out from the mutation sites of the variants, and 101 aa of FPV could be I or T

In the 1980s, CPV-2a and CPV-2b were distinguished using monoclonal antibodies (MAbs) [16, 21]. CPV-2c, the third variant, was identified in Italy in 2000 [17]. The mutation sites of the variants have been mapped for VP2 [22–24]. The mutated amino acid (aa) residues for CPV-2a include M87L, I101T, A300G, D305Y, and V555I; CPV-2b features an I555V reversion and N426D; and CPV-2c features N426E and S297A. Nevertheless, after checking all of the VP2 sequences (a total of 1119 sequences have the collection date and the 555 aa residue) in GenBank, only two isolates (accession numbers M24000 and M24003) feature I at the aa 555 position of VP2 (S1 Table). The two unique viruses with 555I were isolated in 1983 and 1984 when CPV-2 was first identified [25]. According to the circulating type 2a sequences, previous studies concluded that CPV-2a has an I555V reversion mutation due to viral evolution [24, 26]. This finding demonstrated that V555I is not the universal mutation site of CPV-2a and that CPV-2b does not represent an I555V reversion but rather maintains the original V at position 555. Therefore, in this study, the CPV-2a mutations were remapped as 87L, 101T, 300G and 305Y; the CPV-2b mutation was remapped as 426D; and the CPV-2c mutations were remapped as 426E and 297A (Table 1). Except two 555 I viruses, other viruses are 555V which means the VP2 555 aa may need rule out from the mutation sites of the variants.

**Table 1:** Statistics of the mutation sites and the types and rates of mutation in the CPV-2 VP2 gene. Grey shading indicates the key mutations of each variant.

First isolate time^1^	First isolate accession number	Virus type		Amino acid VP2											
				80	93	103	323	564	568	87	101	300	305	426	297
		FPV		K	K	V	D	N	A	M	I/T	A	D	N	S
1978	M38245	Original CPV-2		R	N	A	N	S	G	M	I	A	D	N	S
1980	DQ340404	CPV-2a		R	N	A	N	S	G	L	T	G	Y	N	S
2010	JX475246		CPV-2a-300D	R	N	A	N	S	G	L	T	D	Y	N	S
1990	DQ340411		CPV-2a-297A	R	N	A	N	S	G	L	T	G	Y	N	A
2005	EF599098		CPV-2a-297A300D	R	N	A	N	S	G	L	T	D	Y	N	A
1985	DQ340409	CPV-2b		R	N	A	N	S	G	L	T	G	Y	D	S
1995	KF373568		CPV-2b-297A	R	N	A	N	S	G	L	T	G	Y	D	A
2010	JX475261		CPV-2b-297A300D^2^	R	N	A	N	S	G	L	T	D	Y	D	A
1997	FJ005196	CPV-2c		R	N	A	N	S	G	L	T	G	Y	E	A

^1^, According to the collection date or publication references of GenBank sequences.

^2^, None from dogs. Two isolates (JX475261 and JX475262) from Procyon lotor and one isolate (AB054224) from a cat.

As previous reports, VP2 residue 375 was N in most original CPV-2 isolates and was D in FPV and others CPV-2 variants [24, 27, 28]. However, after checking all of the VP2 sequences (a total of 152 FPV sequences and 1270 CPV-2 sequences have the collection date and the 375 aa residue) in GenBank, FPV and all CPV-2 variants feature D or N at the aa 375 position of VP2 (S1 Table). Therefore, The VP2 375 aa may need rule out from the mutation sites of the variants.

For FPV, after checking 245 VP2 sequences of FPV, only 13 sequences feature I and 220 sequences feature T at the aa 101 position (S1 Table). Hence, the feature of aa 101 position should be updated to be I or T instead of only I in existing rule (Table 1).

### The optional nomenclature of the variants

**Fig 1:**
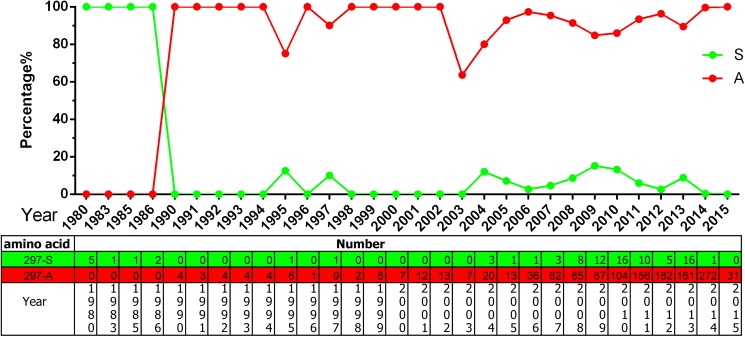
The aa percentages and numbers at the VP2 297 site.

With evolution, further mutations were detected based on CPV-2a and CPV-2b, such as new CPV-2a and new CPV-2b with a S297A [18, 29, 30] mutation and CPV-2c(a) and CPV-2c(b) with a Gly300Asp mutation [19, 20]. The nomenclature of these variants is confusing. In addition, as viral evolution progresses, the naming will continue to be unsustainable. Here, except original CPV-2, the CPV-2a\2b\2c were defined to be the three unique variants, and the following mutation variants are concluded to be sub-variants to CPV-2a\2b: CPV-2a-297A, CPV-2a-300D, CPV-2a-297A300D, CPV-2b-297A, and CPV-2b-297A300D (Table 1). Therein, there are no single mutation sub-variant CPV-2b-300D viruses in the CPV-2b group. There are only three isolates that belong to sub-variant CPV-2b-297A300D, which includes two isolates (JX475261 and JX475262) from *Procyon lotor*, one isolate (AB054224) from a cat, and none from dogs. After summarizing all of the strains with the 297A mutation, we found that since 1990, this mutation was predominant in parvoviruses (Fig 1). If there is a new sit mutation, it could be add into this sustainable nomenclature as a new sub-variant. Only original CPV-2, CPV-2a, CPV-2b, CPV-2c were defined as variants may be a optional nomenclature for CPV research.

### The global distribution of CPV-2a\2b\2c

**Fig 2:**
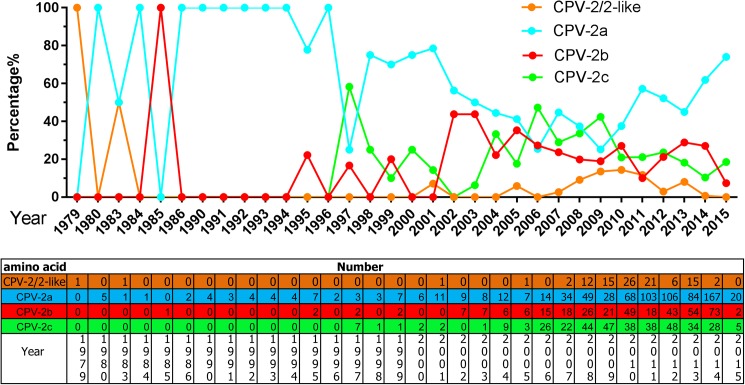
The percentages and numbers of original CPV-2/CPV-2-like, CPV-2a, CPV-2b, and CPV-2c.

**Fig 3:**
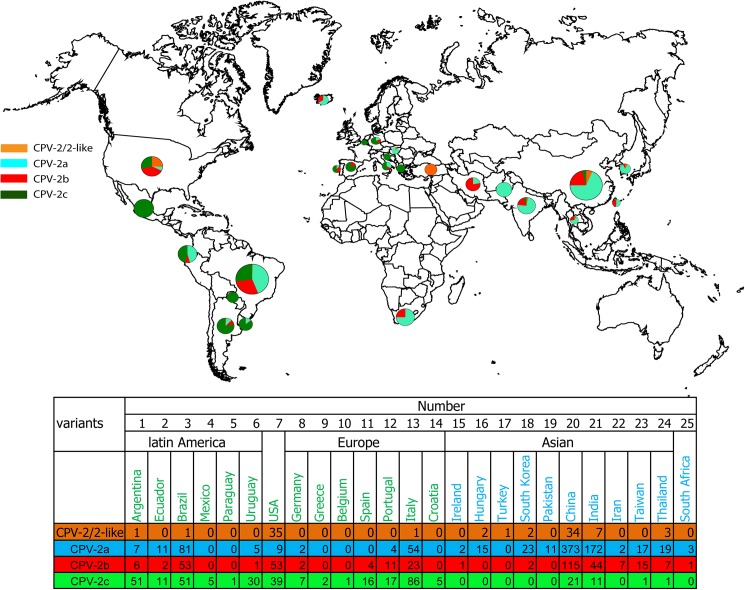
The global distribution of CPV-2a\2b\2c.

As is commonly known, over only a few years in the early 1980s, CPV-2a rapidly and completely replaced original CPV-2 [15]. After CPV-2b and CPV-2c were reported in 1991 and 2001, respectively, it was determined that CPV-2a\2b\2c was globally distributed in the canine population. However, from 2009 to 2015, CPV-2a seemed to become the more predominant strain compared to CPV-2b\2c (Fig 2). Additionally, there are 25 countries that submitted CPV-2 sequences to Genbank (Fig 3), which means CPV-2 globally distributed. However, after analyzing the country distribution of the virus variants, there are some different distribution status in different regions. In Asia, CPV-2a is the predominant variant. CPV-2c which first reported in Italy is the predominant variant in Europe and Latin America (Fig 3).

### CPV-2-like strains may evolve from vaccine strains

Currently, CPV-2a\2b\2c are globally distributed in the canine population. Worldwide, there are also some sporadic identifications of the original CPV-2 [31–34], which were regarded as vaccine strains in the previous study [31]. In this study, eighty samples presented some common aa residues with the original CPV-2 (Table 2). The aa residues of twenty-four CPV-2 strains are similar to the vaccine strains (original CPV-2) and were regarded as vaccine strains, similar to a previous study[31].

**Table 2:** Characteristics of CPV-2 and CPV-2-like strains.

Number	Accession number		Year	Amino acid sites				
				426	87	101	300	305
1	GU212790	Vaccine strain	2009	N	**M**	**I**	**A**	**D**
2	GU212791	Vaccine strain	2009	N	**M**	**I**	**A**	**D**
3	GU212792	Vaccine strain	2009	D	**M**	**I**	**A**	**D**
4	EU659116	Original CPV-2	1979	N	**M**	**I**	**A**	**D**
5	GU569943	Original CPV-2	1983	N	**M**	**I**	**A**	**D**
6	FJ197846	Original CPV-2	2007	N	**M**	**I**	**A**	**D**
7	FJ197847	Original CPV-2	2007	N	**M**	**I**	**A**	**D**
8	GU392236	Original CPV-2	2009	N	**M**	**I**	**A**	**D**
9	GU392237	Original CPV-2	2009	N	**M**	**I**	**A**	**D**
10	GU392239	Original CPV-2	2009	N	**M**	**I**	**A**	**D**
11	GU392240	Original CPV-2	2009	N	**M**	**I**	**A**	**D**
12	GU392241	Original CPV-2	2009	N	**M**	**I**	**A**	**D**
13	GU392242	Original CPV-2	2009	N	**M**	**I**	**A**	**D**
14	GU392243	Original CPV-2	2009	N	**M**	**I**	**A**	**D**
15	GU392244	Original CPV-2	2009	N	**M**	**I**	**A**	**D**
16	KF803602	Original CPV-2	2010	N	**M**	**I**	**A**	**D**
17	KM083036	Original CPV-2	2010	N	**M**	**I**	**A**	**D**
18	KM083037	Original CPV-2	2010	N	**M**	**I**	**A**	**D**
19	KM083038	Original CPV-2	2010	N	**M**	**I**	**A**	**D**
20	KM083039	Original CPV-2	2010	N	**M**	**I**	**A**	**D**
21	KM083040	Original CPV-2	2010	N	**M**	**I**	**A**	**D**
22	KM083041	Original CPV-2	2010	N	**M**	**I**	**A**	**D**
23	KJ170678	Original CPV-2	2010	N	**M**	**I**	**A**	**D**
24	KJ170679	Original CPV-2	2010	N	**M**	**I**	**A**	**D**
25	KJ170681	Original CPV-2	2010	N	**M**	**I**	**A**	**D**
26	KJ194462	Original CPV-2	2010	N	**M**	**I**	**A**	**D**
27	KJ194463	Original CPV-2	2010	N	**M**	**I**	**A**	**D**
28	KP406929	Original CPV-2	2013	N	**M**	**I**	**A**	**D**
29	KP406930	Original CPV-2	2013	N	**M**	**I**	**A**	**D**
30	FJ222824	CPV-2-like	2005	N	L	T	**A**	Y
31	FJ435342	CPV-2-like	2008	N	**M**	**I**	**A**	V
32	FJ435344	CPV-2-like	2008	N	**M**	**I**	**A**	V
33	FJ432718	CPV-2-like	2008	N	**M**	**I**	**A**	V
34	FJ435348	CPV-2-like	2008	N	**M**	**I**	**A**	V
35	KJ186140	CPV-2-like	2008	N	**M**	T	I	**D**
36	EU441280	CPV-2-like	2008	N	L	T	**A**	**D**
37	GU392238	CPV-2-like	2009	N	**M**	T	G	**D**
38	JN867599	CPV-2-like	2009	N	L	T	D	**D**
39	JN867600	CPV-2-like	2009	N	L	T	D	**D**
40	KF803590	CPV-2-like	2010	N	**M**	**I**	G	Y
41	KF803594	CPV-2-like	2010	N	**M**	**I**	G	Y
42	KF803600	CPV-2-like	2010	N	**M**	**I**	G	Y
43	KF803601	CPV-2-like	2010	N	**M**	**I**	G	Y
44	KF803603	CPV-2-like	2010	D	**M**	**I**	G	Y
45	JX475246	CPV-2-like	2010	N	L	T	D	**D**
46	JX475258	CPV-2-like	2010	N	L	T	D	**D**
47	JN867598	CPV-2-like	2010	N	L	T	D	**D**
48	JX475231	CPV-2-like	2011	N	L	T	D	**D**
49	JX475233	CPV-2-like	2011	N	L	T	D	**D**
50	JX475234	CPV-2-like	2011	N	L	T	D	**D**
51	JX475235	CPV-2-like	2011	N	L	T	D	**D**
52	JX475239	CPV-2-like	2011	N	L	T	D	**D**
53	JX475248	CPV-2-like	2011	N	L	T	D	**D**
54	JX475255	CPV-2-like	2011	N	L	T	D	**D**
55	JX475264	CPV-2-like	2011	N	L	T	D	**D**
56	JX475265	CPV-2-like	2011	N	L	T	D	**D**
57	JX475268	CPV-2-like	2011	N	L	T	D	**D**
58	JX475271	CPV-2-like	2011	N	L	T	D	**D**
59	JX475279	CPV-2-like	2011	N	L	T	D	**D**
60	JX475280	CPV-2-like	2011	N	L	T	D	**D**
61	JX475281	CPV-2-like	2011	N	L	T	D	**D**
62	JX475282	CPV-2-like	2011	N	L	T	D	**D**
63	JX475284	CPV-2-like	2011	N	L	T	D	**D**
64	JX475285	CPV-2-like	2011	N	L	T	D	**D**
65	JX475286	CPV-2-like	2011	N	L	T	D	**D**
66	JX475287	CPV-2-like	2011	N	L	T	D	**D**
67	JX475288	CPV-2-like	2011	N	L	T	D	**D**
68	KM236572	CPV-2-like	2012	N	**M**	**I**	I	**D**
69	KJ813890	CPV-2-like	2012	N	L	T	D	**D**
70	KF539801	CPV-2-like	2012	N	L	T	G	**D**
71	KF539803	CPV-2-like	2012	N	L	T	G	**D**
72	KC262178	CPV-2-like	2012	N	L	T	**A**	**D**
73	KJ813831	CPV-2-like	2013	N	L	T	D	**D**
74	KJ813832	CPV-2-like	2013	N	L	T	D	**D**
75	KJ813833	CPV-2-like	2013	N	L	T	D	**D**
76	KJ813834	CPV-2-like	2013	N	L	T	D	**D**
77	KJ813835	CPV-2-like	2013	N	L	T	D	**D**
78	KJ813836	CPV-2-like	2013	N	L	T	D	**D**
79	KJ813837	CPV-2-like	2013	N	L	T	D	**D**
80	KJ813870	CPV-2-like	2013	N	L	T	D	**D**
81	KP406926	CPV-2-like	2013	N	**M**	**I**	G	Y
82	KP641336	CPV-2-like	2013	N	L	T	**A**	**D**
83	KP641337	CPV-2-like	2013	N	L	T	**A**	**D**
84	KP641338	CPV-2-like	2013	N	L	T	**A**	**D**
85	KP071953	CPV-2-like	2014	D	**M**	**I**	G	Y

Bold presents a similar residue for the vaccine strains.

However, fifty-six strains (CPV-2-like) show one or several mutation sites similar to the vaccine strains (Table 2). Currently, the modified live virus vaccines, which are based on the original CPV-2, are widely used. In fact, after a dog is vaccinated, the modified live virus is able to replicate in the animal’s intestinal mucosa and can be shed in the feces [35, 36]. Although the attenuated CPV-2 vaccine strains are stable in limited passages in the dog (i.e., five or six passages) [37, 38], original CPV-2 or CPV-2-like strains were identified from sick and vaccinated dogs [34, 36]. Although there is no way to rule out PCR errors in sequences retrieved from the public database, any possibility of reversion to virulence among the vaccine strains needs to be taken seriously.

### The evolution and mutational tendency of CPV-2 VP2

**Fig 4:**
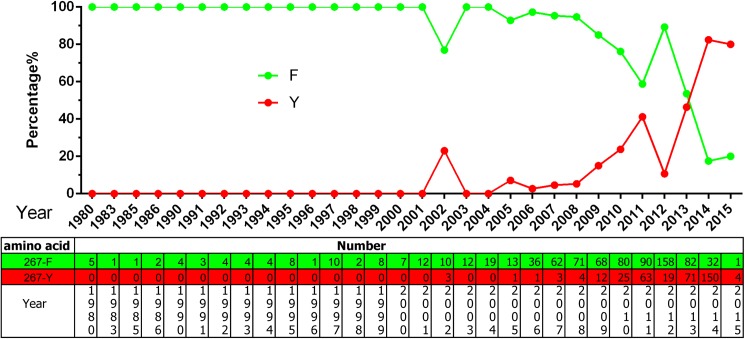
The aa percentages and numbers at the VP2 267 site.

**Fig 5:**
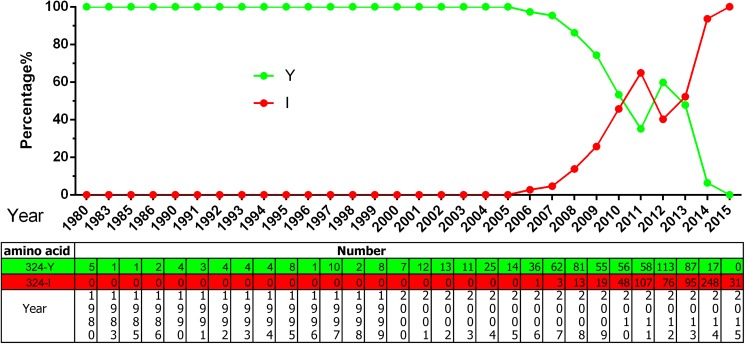
The aa percentages and numbers at the VP2 324 site.

**Fig 6:**
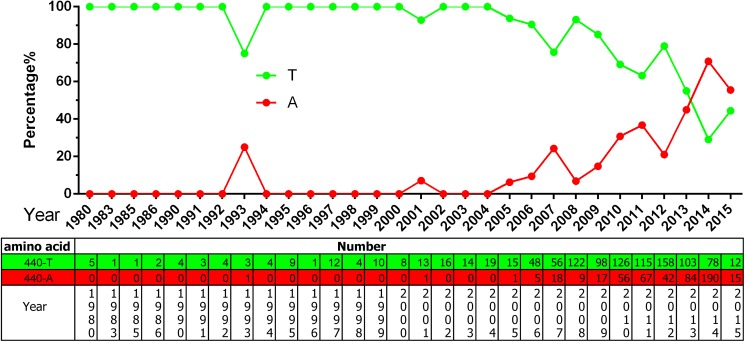
The aa percentages and numbers at the VP2 440 site.

According to an analysis of VP2 genes from all isolates, another three aa residues have become new potential mutation foci: F267Y, Y324I, and T440A. Strains encoding 267Y first appeared in 2002 and have become predominant since 2014 (Fig 4). Strains encoding 324I first appeared in 2006 and have become more common, also becoming predominant in 2014 (Fig 5). From Fig 5 it can be deduced that the Y324I substitution progresses perfectly from 2006 to 2011. Strains encoding 440A first appeared in 1993, but the variant appeared continuously from 2005 (Fig 6). Although there is no way to rule out PCR errors in sequences retrieved from the public databases, the tendency of aa substitution is definitely clear in terms of the figs.

**Fig 7:**
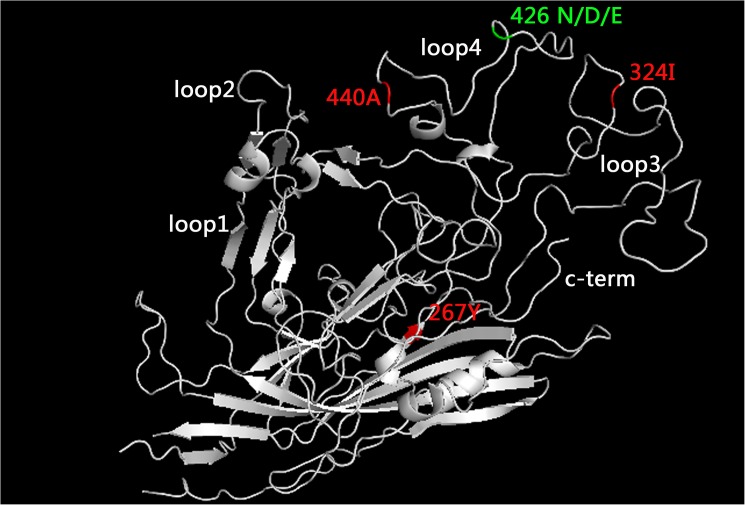
A structure model of VP2 and the distribution of the new aa mutation sites (267, 324, 440). These locations were visualized using the PyMOL Molecular Graphics System, version 1.5.0.4 Schrödinger, LLC.

The VP2 structure was downloaded from SWISS-MODEL and were depicted in PyMOL (Version 1.5.0.4). The aa 267 residue does not locate in any loop (Fig 7). The aa 324 residue is located in loop 3 (Fig 7), whose top protrusion site consists of aa 300 to 303 [39], indicating that 324 is not in the top protrusion. In contrast, the aa 440 residue is located in loop 4 (Fig 7), whose top protrusion sites consists of aa 422 to 428 and 433 to 443 [39], indicating that 440 is in the top protrusion of loop 4.

## Discussion

The VP2 V555I mutation was formerly defined as one CPV-2 mutation site that was important for CPV-2a evolution. The VP2 aa 375 of CPV-2 feature N in most original CPV-2 isolates and feature D in FPV and others CPV-2 variants. Here, after analyzing all VP2 sequence of CPV-2 and FPV(from 1978 to 2015), the VP2 amino acid 555 and 375 positions of CPV-2 were first ruled out as a universal mutation site in CPV-2a and amino acid 101 position of FPV feature I or T instead of only I in existing rule. the VP2 V555I was first ruled out from the universal CPV-2a mutation sites. The previous definition may be due to the limited number of sequences available from that period. The nomenclature of CPV-2 variants is inconsistent and confusing. Here, the original CPV-2 and CPV-2a\2b\2c were defined to be the four unique variants that is a optional nomenclature would serve CPV-2 research.

CPV-2a is the predominant variant in Asia and CPV-2c is the predominant variant in Europe and Latin America, which remind different regions may be need pay more attention to the predominant variant. There are some sporadic identifications of the original CPV-2 and CPV-2-like strains. The original CPV-2 were regarded as vaccine strains and CPV-2-like strains have been identified from sick and vaccinated dogs [31, 34]. Therefore, CPV-2-like strains may be the result of the reversion of vaccine strains to virulence.

With antigenic drift, three new mutations at VP2 (F267Y, Y324I, and T440A) have occurred. Recently, inactivated vaccines and modified live virus vaccines have become widely used [24]. However, there are some concerns regarding the complete efficacy of existing vaccines against the antigenic variants [40–43]. Because viruses always evolve to escape the immune system via antigenic drift, the new mutations (F267Y, Y324I, and T440A) may be caused by vaccine immune pressure. Meanwhile, these same new mutations (F267Y, Y324I, and T440A) may be responsible for vaccine failure. The aa 440 residue located in the top protrusion of loop 4 of the VP2 protein. The aa 426 residue that is located in one top protrusion of loop 4 has been defined as the major mutation site for CPV-2 evolution. Mutation of the aa 440 residue that is located in the other top protrusion of loop 4 may cause viral antigenic drift. Of note, the T440A mutation has been reported in previous studies [44, 45]. Thus, strains with VP2 440A may become a novel CPV sub-variant. Certainly, the functional or other testing of the mutants needs to be further performed.

Finally, this sequence summary reveals a new perspective regarding CPV-2 evolution and represents a significant way to discover new characteristics and to overcome the problems arising from periods with limited available sequencing data.

## Supporting information

S1 Table The number of different amino acid of 375, 555 and 101375 and 555 sites in different CPV-2 variants and FPV. (DOCX)
