# Supplementary material for: The genetic evolution of canine parvovirus – A new perspective
Source: PLoS One. 2017 Mar 31;12(3):e0175035. doi: 10.1371/journal.pone.0175035 (PMC5376324; doi:10.1371/journal.pone.0175035)
Supplement: S1 Table — (DOCX) [file pone.0175035.s001.docx]

| **Table S1** The number of different amino acid of 375, 555 and 101 sites in different CPV-2 variants and FPV. | | | | | | | | | | | |  |
| --- | --- | --- | --- | --- | --- | --- | --- | --- | --- | --- | --- | --- |
| Variant | **375** | |  | **555** | |  | **101** | | | | |  |
|  | **N** | **D** |  | **V** | **I** |  | **I** | **T** | | | |  |
| FPV | 1 | 151 |  | 132 | 0 |  | 13 | 220 | | | |  |
| Original CPV-2 | 5 | 20 |  | 29 | 0 |  | 29 | 0 | | | |  |
| CPV-2a | 8 | 737 |  | 516 | 2 |  | 0 | 468 | | | |  |
| CPV-2b | 4 | 245 |  | 250 | 0 |  | 0 | 181 | | | |  |
| CPV-2c | 0 | 251 |  | 324 | 0 |  | 0 | 231 | | | |  |
|  |  |  |  |  |  |  | | |  |  |  | |
